# Tissue culture and genetic transformation of cabbage (*Brassica oleracea* var. *capitata*): an overview

**DOI:** 10.1007/s00425-018-2961-3

**Published:** 2018-07-31

**Authors:** Aneta Gerszberg

**Affiliations:** Department of Genetics, Plant Molecular Biology and Biotechnology, Faculty of Biology and Environmental Protection, University of Lodz, Banacha 12/16, 90-237 Lodz, Poland

**Keywords:** *Brassica oleracea* var*. capitata*, Cabbage, Tissue culture, Genetic transformation

## Abstract

The main goal of this publication is an overview of the biotechnological achievements concerning in vitro cultures and transformation of *Brassica oleracea* var. *capitata*.

Faced with the requirements of the global food market, intensified work on the genetic transformation of economically important plants is carried out in laboratories around the world. The development of efficient procedures for their regeneration and transformation could be a good solution for obtaining, in a shorter time than by traditional methods, plants with desirable traits. Furthermore, conventional breeding methods are insufficient for crop genetic improvement not only because of being time-consuming but also because they are severely limited by sexual incompatibility barriers. This problem has been overcome by genetic engineering, which seems to be a very good technique for cabbage improvement. Despite the huge progress that has been made in the field of plant regeneration and transformation methods, up to now, no routine transformation procedure has been developed in the case of cabbage. This problem stems from the fact that the efficiency of cabbage transformation is closely related to the genotype and some varieties are recalcitrant to transformation. It is obvious that it is not possible to establish one universal regeneration and transformation protocol for all varieties of cabbage. Therefore, it seems fully justified to develop the above-mentioned procedures for individual economically important cultivars. Despite the obstacles of cabbage transformation in laboratories of many countries, especially those where this vegetable is extremely popular (e.g., China, India, Korea, Malaysia, Pakistan), such attempts are made. This article reviews the achievements in the field of tissue culture and cabbage transformation from the last two decades.

## Introduction

**Fig. 1:**
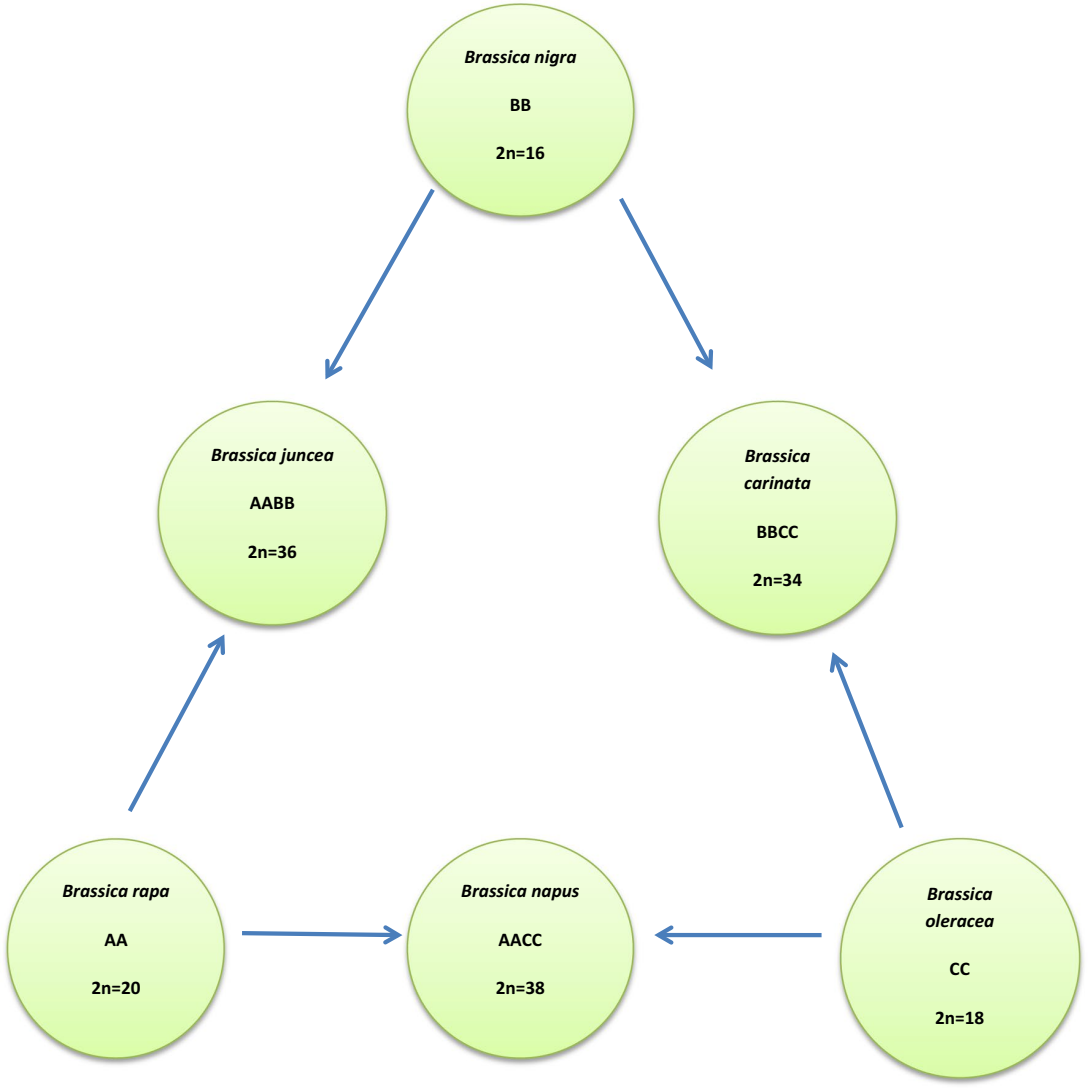
Relationships between members of the plant genus of *Brassica* (the U triangle) (based on Liu et al. 2014)

Among large genus of *Brassica*, six species (*Brassica rapa, B. nigra, B. oleracea, B. carinata, B. juncea, B. napus*) are widely used worldwide as forage, oil seeds, condiments or vegetable crops. Three of them (*B. nigra, B. rapa and B. oleracea*) are diploid, while the other three are allotetraploids (Fig. 1). The extensive research confirmed their mutual genetic relationships (Liu et al. 2014).

One of the most important components of the human diet alongside fruits is vegetables. Among vegetables, one of the most important groups, due to their nutritional value, are those belonging to the *Brassicaceae* family (2n = 18) (Fig. 1). It is a multifarious group that includes crops such as cauliflower, cabbage, Savoy cabbage, collards, Brussels sprouts, broccoli, turnip, rutabaga, kale and kohlrabi (Ravanfar et al. 2017). *Brassica oleracea* var*. capitata* (common name: cabbage) is an extremely valuable source of folic acid, vitamins (e.g., C, K, A), flavonoids and calcium (Gerszberg et al. 2015). Moreover, cabbage comprises secondary metabolites (glucosinolates) and amino acids that contribute to its anti-inflammatory and anticancer properties (Hafidh et al. 2013; Zielińska et al. 2015). The largest producers of cabbage in the world are China, India and Pakistan, while Poland ranks among the top ten (FAOSTAT 2016). The biotic and abiotic stresses as well as pests have a significant impact on the loss of both quality and quantity of cabbage yield (Yi et al. 2013). Mostly, severe damages are caused by fungal diseases caused by *Alternaria brassicicola*, *Botrytis cinerea*, *Pythium* spp. *Plasmodiophora brassicae* as well as by lepidopteran insects (*Plutella xylostella*) (Yi et al. 2013; Gerszberg et al. 2015). To overcome this problem, many studies have been carried out to optimize the regeneration (Gerszberg et al. 2015; Daud et al. 2015; Gambhir et al. 2017a, b) and transformation procedures of *B. oleracea* var. *capitata* (Yi et al. 2013; Hur and Min 2015; Ravanfar et al. 2017) (Table 1). Since conventional breeding techniques are inadequate, it seems that the genetic transformation of cabbage appears to be an important method for improving this vegetable. The advancement of genetic engineering can overcome the barriers associated with the sexual incompatibility of *Brassica oleracea* cultivars, resulting in hybrids with new desirable agronomic traits. It is worth noting that many articles refer to the genetic modification of rape or cauliflower, while relatively few publications focus strictly on the transformation of cabbage. Therefore, the objective of this article is to summarize the achievements in the field of regeneration and genetic transformation of cabbage.

**Table 1:** Examples of successful *B. oleracea* var. *capitata* regeneration

Cultivar	Type of explants	The best variant of medium	% of shoot regeneration	References
Baochun F1	Mesophyll protoplast culture	MS + 3 mg/L kin + 0.1 mg/L GA_3_	100% callus produced shoots	Fu et al. (1985)
Varaždinsko, Hawke F2	Microspore culture	NLN + 5 mg/L ABA-germination embryos B5 + 2% sucrose	54.7–70.6%	Rudolf et al. (1999)
Zigan No.1, Hongmu, Ruby ball	Protoplast culture	In first stage of protoplast culture, it is important to add nurse cell of tuber mustard. MS + 1 mg/L BA + 0.2 mg/L NAA—regeneration of plantlets	33% Zigan No.1 47% Hongmu 56% Ruby ball	Chen et al. (2004)
Křimické Holt Landrace Zakamenné Landrace Zázrivá Landrace Lutiše Trvanlivé D Vysocké Kalibos	Cotyledonary embryos	MS + 1% sucrose + 1% agar	18.18 and 16.6% 5.88 and 11.11% 23.53 and 39.34% 44.83 and 52.00% 21.74% 42.86% 25.93% 71.79%	Klima et al. (2004)
Kamienna głowa Sława of Einkhunzen	Anther culture	B5 + 20 mg/L Kin + 2% sucrose	Kamienna głowa 17.1% Sława z Einkhunzen 8.3%	Krzyżanowska et al. (2006)
N/A	Hypocotyl, cotyledon	MS + 2 mg/L BA + 0.1 mg/LNAA	50% (hypocotyls) 40% (cotyledons)	Munshi et al. (2007)
Rubin	Cotyledon, hypocotyl, root	MS + 1.0 mg/L + 0.1 mg/L IBA Kin (cotyledon) MS + 1 mgL/BA (hypocotyl) MS + 1mgL/BA + 0.1mg/L IBA(root)	41% (cotyledon) 75%(hypocotyl) 14% (root)	Pavlovic et al. (2010)
DL 20, DM 56	Microspore culture	MS + 8.9 BAP µM/L + 2.7 NAA µM/L + 50 µM/L	42.9% (DL 20), 40.1% (DM 56)	Cristea et al. (2012)
Kamienna głowa, Amager, Kilaxy F1, Benia F1, Reball F1, Rodeo F1	Protoplasts from leaves and hypocotyls	MS medium PGRs free	Mean 0.0–34.6% 29.8% for leaves-derived protoplasts 26.2% for hypocotyl-derived protoplasts	Kielkowska and Damaus (2012)
R1, R5, R7, R9, R33, K1, K6, K7, K23, K29, K35, K48, K50, K75	Lateral buds	MS + 2 mg/L BA + 1.0 IBA mg/L	80–100%	Pavlovic et al. (2012)
Cabeza Negra 2, Arena, Red Amager	Cotyledon, hypocotyl, stem	MS + 2 mg/L BAP + 0.2-04 mg/L IBA or NAA (hypocotyl)	300 shoots (Cabeza Negra 2 and Red Amager) (hypocotyl) 25 shoots (Arena)	Dănăilă-Guidea et al. (2012)
K7	Zygotic embryos	MS + 1 mg/L BA or MS + 1 mg/L Kin	56%	Pavlovic et al. (2013)
Pride of India	Cotyledon, hypocotyl	MS + 1.5 mg/L BAP + 0.5 mg/L NAA (cotyledon) MS + 2 mg/L BAP + 0.25 mg/L IAA	5.334 shoots per explant (cotyledon) 6.0 shots per explant (hypocotyl)	Sharma et al. (2014)
KY cross	Cotyledon, hypocotyl	MS + 2.27 µM TDZ (hypocotyl) MS+	56.67% (cotyledon) 80% (hypocotyl)	Ravanfar et al. (2014)
Saint	Hypocotyl	MS + BAP 4 mg/l + IAA 5 mg/l + Zeatin 4 mg/l	100%	Qamar et al. (2014)
Kamienna głowa, Amager, Sława of Einkhunzen, Brunświcka, Ditmarska, Zora, Ula, Replika	Cotyledon, hypocotyl	MS + 8.88 μM (BAP + 0.53 μM NAA)	22.2–66.6% (hypocotyl)	Gerszberg et al. (2015)
N/A	Stem, root, leaf, petiole	MS + 1.0 mg/L NAA + 1.5 mg/L BAP (steam)	93.18% (stem) 91.40% (root)	Daud et al. (2015)
Zhonggan 11, Chunfeng, Parel, Meiweizaosheng	Microspore culture	NLN + 20 mg/L AsA	Zhonggan 11 164.2 (no.) Chunfeng 15.1 Parel 53.1 Meiweizaosheng 57.5	Zeng et al. (2015)
Pride of India	Leaf, petiole	MS + 0.22 mg/L TDZ + 0.02 mg/L NAA (leaf) MS + 0.33 mg/L TDZ + 0.02 mg/L NAA (petiole)	91.11% (leaf) 88.88% (petiole)	Gambhir and Srivastava (2015)
Ercis	Cotyledon, hypocotyl	MS + 2 mg/L BAP (hypocotyl) MS + 2 mg/l BAP + 0.1 mg/l NAA and MS + 0.5 mg/l BAP (cotyledon)	100% (hypocotyl) 91.6% (cotyledon)	Ertac and Tuncer (2016)
Pride of India	Cotyledon, hypocotyl	MS + 0.330 mg/l TDZ + 79.70 mg/l IAA (cotyledon) MS + 0.220 mg/l TDZ + 0.088 mg/l IAA (hypocotyl)	91.11% (cotyledon) 94.4% (hypocotyl)	Gambhir et al. (2017a)

*N/A* not available

## Tissue culture of *Brassica oleracea* var*. capitata*

Establishment of an efficient regeneration protocol is a prerequisite step for transferring genes into plants. Plant tissue culture research in cabbage was reported by different scientists exploiting various explants such as mesophyll protoplast culture (Fu et al. 1985; Chen et al. 2004), microspore cultures (Rudolf et al. 1999), cotyledons and hypocotyls (Klima et al. 2004; Munshi et al. 2007; Pavlovic et al. 2010; Ravanfar et al. 2014; Sharma et al. 2014; Gerszberg et al. 2015; Gambhir et al. 2017a), meristematic apex (Dănăilă-Guidea et al. 2012), roots (Pavlovic et al. 2010; Daud et al. 2015), lateral buds (Pavlovic et al. 2012), androgenic embryos (Krzyżanowska et al. 2006), immature zygotic embryos (Pavlovic et al. 2013), leaf and petiole (Gambhir and Srivastava 2015). Considering cabbage, many results pointed out that this crop regeneration and transformation efficiency greatly depended on the genotype (Gerszberg et al. 2015). Thus, it is fully justified to conduct studies on regeneration potential of different cultivars of *B. oleracea* var. *capitata*.

### Type of explants

To develop the best regeneration protocol for cabbage, different factors such as the type of explant, its age, media supplemented with various plant growth regulators (PGRs) were examined. Recently, research was focused mainly on hypocotyl and cotyledon explants pointing to their huge potential for shoot organogenesis (Munshi et al. 2007; Gerszberg et al. 2015; Gambhir et al. 2017a). Munshi et al. (2007) and Sharma et al. (2014) proved that cotyledon explants showed organogenesis superiority over hypocotyl ones. Nevertheless, a lot of studies reported that better regeneration effects were obtained using hypocotyl explants (Pavlovic et al. 2010; Ravanfar et al. 2014; Gerszberg et al. 2015). However, the subtypes of hypocotyl explants (parts of a hypocotyl section) have no effect on the regeneration of shoots (Gerszberg et al. 2015). Additionally, morphogenic response in hypocotyl explants occurred faster than the cotyledon ones (Ravanfar et al. 2014). Several studies compared quite different explants. For example, Daud et al. (2015) showed that the efficiency of shoot regeneration ability followed the order stem > root > petiole > leaf. Generally in most species, including cabbage, not only the type of explant but also its age is important. Based on literature data, it is assumed that explants from 3- to 5-day-old seedlings gave the best regeneration results among various *Brassica* spp. (rev. Cardoza and Stewart 2004). Considering the young age of explants, it should be emphasized that their physiological and biochemical status is very active which means that the cell wall is not so rigid. As a consequence, it is more susceptible to the impact of environmental factors (e.g., exogenous PGRs). In fact, such young explants are too small for convenient manipulation. Probably that is why the majority of researchers use much older seedlings, 7- or even 10-day-old (Pavlovic et al. 2010; Ravanfar et al. 2014; Gerszberg et al. 2015; Gambhir et al. 2017a).

### Types of media and growth regulators

The choice of an appropriate medium and the right dose of PGRs is another crucial factor for cabbage efficient regeneration. So far, many variants of substrate based on MS (Murashige and Skoog, 1962) or B5 (Gamborg et al. 1968) have been tested (Krzyżanowska et al. 2006; Rafat et al. 2010; Ravanfar et al. 2014; Gerszberg et al. 2015; Gambhir et al. 2017a). The addition of hormones to the substrate was not always necessary. For example, in the case of cabbage regeneration from androgenic embryos, B5 phytohormone free medium proved better in comparison to MS medium (Krzyżanowska et al. 2006). However, medium supplementation with exogenous PGRs has a significant impact on callus inductions, morphogenesis and rooting. A lot of publications indicated that addition of cytokinins alone or with auxins at low concentration significantly increased the efficiency of cabbage regeneration (Krzyżanowska et al. 2006; Pavlovic et al. 2013; Gambhir and Srivastava 2015; Gerszberg et al. 2015; Ertac and Tuncer 2016). At present, researchers have a range of exogenous PGRs to be used in plant in vitro culture. The literature data show that in in vitro cultures for cabbage regeneration, indole-3-acetic acid (IAA), indole-3-butyric acid (IBA), α-naphthylacetic acid (NAA), 2,4-D (2,4-Dichlorophenoxy-acetic acid), 6-benzyloaminopurine (6-BAP), kinetin (Kin), thidiazuron (TDZ), 6-(gamma,gamma-dimethylallylamino)purine (2iP) are most commonly used (Chen et al. 2004; Sharma et al. 2014; Ravanfar et al. 2014; Gerszberg et al. 2015; Gambhir et al. 2017a). In protoplast culture, 2,4-Dichlorophenoxy-acetic acid (2,4-D), picloram, and gibberellic acid (GA_3_) were tested (Fu et al. 1985; Chen et al. 2004; Liu et al. 2007), while in microspore culture abscisic acid (ABA) was used (Rudolf et al. 1999). The modifications of the substrate conditions allowed for successful *B. oleracea* var. *capitata* shoot induction from various explants from different cultivars (Table 1).

Summing up the available results (Table 1), it can be concluded without any doubt that the best results of cabbage regeneration were obtained when MS medium fortified with cytokinins and a small amount of auxins was used. Furthermore, the research results suggested the superiority of one type of cytokinin (TDZ) over the others (BAP, 2iP, Kin etc.). Ravanfar et al. (2014) hypothesized that this resulted from the fact that TDZ was able to induce the synthesis of endogenous auxins. Although the induction of shoots from cotyledons is possible, majority of the studies proved that hypocotyls explant had higher morphogenic potential.

### Organogenesis

To regenerate plantlets in vitro, two methods are exploited: organogenesis (direct and indirect) and embryogenesis (direct or indirect). Considering the goal (e.g., heredity or genetic transformation) of the study conducted, the first method is much more advisable because it allows evasion of genetic variations. Regarding cabbage, there are reports on its regeneration by direct (Gambhir and Srivastava 2015; Gambhir et al. 2017a) and indirect organogenesis (Munshi et al. 2007; Dănăilă-Guidea et al. 2012; Gerszberg et al. 2015; Ertac and Tuncer 2016) as well as by somatic embryogenesis pathway (Pavlovic et al. 2013). It should be emphasized that, although somatic embryogenesis is successfully exploited in a wide range of different species, it has been seldom notified in *Brassicas*. The literature data clearly indicate that the effectiveness of regeneration is significantly correlated with the genotype and the type of explant (Klima et al. 2004; Munshi et al. 2007; Krzyżanowska et al. 2006; Dănăilă-Guidea et al. 2012; Gerszberg et al. 2015). It turned out that there were cultivars recalcitrant to regeneration under the applied conditions (Dănăilă-Guidea et al. 2012; Gerszberg et al. 2015). Additionally, it was noticed that the quantity of callus produced from explants did not positively correlate with the subsequent shoot induction. Moreover, the cotyledon explants produced much smaller callus amount and this process was slower in comparison to the hypocotyl explants (Gerszberg et al. 2015).

Rooting is the last step of plant regeneration in vitro. It depends on many factors including the medium composition, PGRs types and concentrations, as well as the physiological status of plantlets. Different variants of MS or ½ MS medium with or without various types and concentrations of auxin (e.g., IBA, IAA, NAA or 2,4-D), were successfully used to induce cabbage rooting process (Munshi et al. 2007; Sharma et al. 2014; Ravanfar et al. 2014; Qamar et al. 2014; Ertac and Tuncer 2016; Gambhir et al. 2017a). According to some researchers, the best rooting results are obtained using PGR-free medium (Ravanfar et al. 2014). On the other hand, others achieved better results when using MS supplemented with small concentrations of auxins (ranging from 0.1 to 0.5 mg/L) (Munshi et al. 2007; Sharma et al. 2014; Gerszberg et al. 2015; Gambhir et al. 2017a). Thus, additives, even at small concentrations, significantly intensified the process of rooting, while their higher concentrations affected root morphologies (Pavlovic et al. 2010; Gerszberg et al. 2015). Furthermore, Sharma et al. (2014) pointed out that the rooting process depended on the type of auxin, its concentration as well as the plantlet from which the explant came from. Munshi et al. (2007), similar to Sharma et al. (2014), achieved the best rooting results exploiting IBA. Certainly, 2,4-D proved to be the least effective in rooting, which caused the formation of callus at the base of the regenerated plantlets (Munshi et al. 2007). Similar effects were obtained in the case of elevated NAA (5.2–8.0 µM) concentrations (Gerszberg et al. 2015). Pavlovic et al.’s (2010) research indicated that in the case of cabbage, the type of auxin and its concentration in the rooting medium sugar concentration are also important.

### Possible problems during in vitro cultures

During in vitro regeneration of different plant species including cabbage, various problems may arise, including vitrification of plant tissue or chlorosis and necrosis along with prolonged growing period on a given substrate. Vitrification involves excessive hydration of tissues (“glassy” appearance), caused by high levels of moisture in the jar, growth regulators (e.g., BAP) in the nutrient medium as well as limited exposure to light (Pavlovic et al. 2012; Ravanfar et al. 2014). Such plants were characterized with deformed chloroplasts, moreover, they were found to have problems with the synthesis of chlorophyll and other dyes as well as enzymatic activity. As a result, they are not able to live under greenhouse conditions. This problem can be solved with increasing the concentration of the nutrient medium (e.g., agar), thus limiting the availability of water. Chlorosis and then necrosis may be the result of accumulation of ethylene in the jars or Petri dishes due to reduced gas exchange. Another possible explanation of the aforementioned phenomena may be the leakage of phenolic compounds, oxidation of which yields toxic compounds to the substrate. This phenomenon often occurs in in vitro cultures of plants rich in phenolic compounds, and cabbage is such a plant (Gerszberg et al. 2015). Since it is known that silver nitrate (AgNO3) strongly inhibits ethylene action, it can be added to the medium at a low concentration to overcome this obstacle (Cristea et al. 2012).

### Hardening

Subsequently, fully regenerated plants should be hardened. Typically, cabbage plants are placed in the substrate, which is a mixture of soil and compost (2:1) (Munshi et al. 2007), coconut and vermicompost (7: 3) (Ravanfar et al. 2014), soil and perlite (3:1) (Gerszberg et al. 2015) or coco peat (Gambhir et al. 2017a). According to the literature data, the survival rate of regenerated plants after hardening varies from 70 to 95% (Sharma et al. 2014; Daud et al. 2015; Gerszberg et al. 2015). Pavlovic et al. (2010) indicated an interesting correlation between the concentration of sugar in the rooting substrate and the survival of the regenerated plants during acclimation, namely, the higher sugar concentration, and the higher survival of plants.

## Genetic transformation of *Brassica oleracea var. capitata* via *Agrobacterium*

*Agrobacterium*-mediated transformation is widely employed to transfer a gene of interest (GOI) to crop species important from the economic point of view. However, most of them are recalcitrant to genetic modifications, since a lot of protocols are developed for model plants. Therefore, based on existing protocols, they should be properly refined for individual commercial plant species including cabbage.

**Fig. 2:**
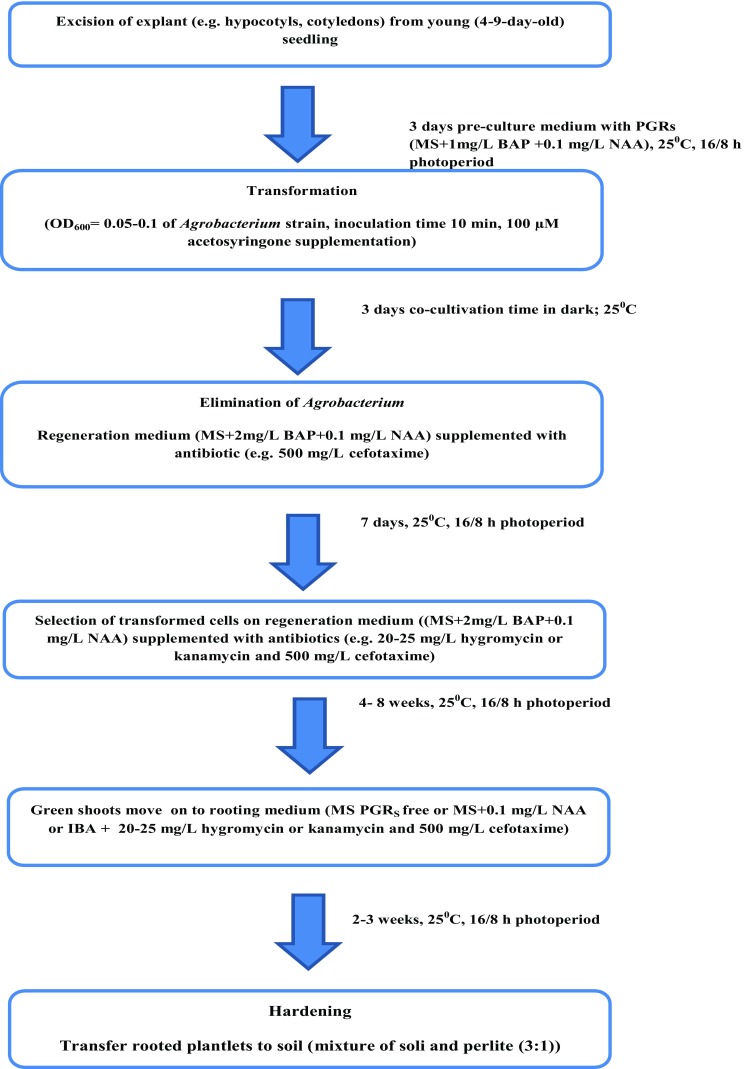
Outline of procedure for *Agrobacterium*-mediated transformation of cabbage

A review of the literature indicates the use of both *A. rhizogenes* and *A*. *tumefaciens* for the transformation of cabbage. The former pathway is rarely used for cabbage transformation. Considering *A. rhizogenes*-mediated transformation, it should be noted that transgenic roots of cabbage were successfully obtained, whereas difficulties appeared at the stage of plant regeneration. The results pointed out that shoot regeneration from hairy roots was genotype dependent and some genotypes were even recalcitrant to the applied regeneration condition (Stretnovic-Rajicic et al. 2006). Moreover, the phenotype of some hairy root-derived plantlets was abnormal which was manifested by reduced apical dominance, wrinkled leaves, short internodes and male sterility (poor pollen production) (Berthomieu and Jouanin 1992; Bhala and Singh 2008). On the other hand, some of the obtained plants were normal in terms of phenotype and even better developed than the control plants (Stretnovic-Rajicic et al. 2006). It can be concluded that due to the described difficulties, *A. rhizogenes*-mediated transformation method is rarely used to transform cabbage. This is also confirmed by scanty publication data concerning this issue. *A. tumefaciens*-mediated transformation is used more often (Yi et al. 2013; Hur and Min 2015). However, also in this case the transformation efficiency is not satisfactory (Liu et al. 2008). Nevertheless, attempts are made to optimize individual transformation parameters (Bhala and Singh 2008; Rafat et al. 2010). Moreover, to reduce public concerns over the exploit of genes giving antibiotic resistance, some research groups tried to establish an alternative, safer selection system (Hur and Min 2015). An effective genetic transformation of cabbage is strictly genotypically dependent; furthermore, some varieties are recalcitrant to transformation. So, it is clear that it is not possible to develop a universal cabbage transformation protocol. Therefore, it seems fully logical to establish a reliable transformation and regeneration procedure for specific cultivars. This trend in studies conducted in many laboratories is observed (Table 2). Most studies are dedicated to introducing genes conferring tolerance to insect or abiotic stresses. Based on the available literature in this topic, the current article proposes an outline of the general procedure for cabbage transformation via *Agrobacterium* (Fig. 2). It may be a starting point for modification and developing a protocol dedicated to a specific variety.

**Table 2:** Genetic improvements of *Brassica oleracea var. capitata*

*B. oleracea var. capitata* cultivar	Technique of gene transfer	Gene transfer	Improvement in traits	Type of explant used	References
161	*A. rhizogenes* (A_4_K, A_4_H)	*nptII*, *hph*	Resistance to kanamycin, hygromycin	Leaf petioles, internodes	Berthomieu and Jouanin (1992)
King Cole	*A. tumefaciens* (AB1)	*cry1a (c)*	Resistance to diamond back moth (*P. xylostella*)	Hypocotyls, cotyledons, petioles, peduncle from flowering stalks	Metz et al. (1995)
Yingchun, Jingfeng	*A. tumefaciens* (LBA4404)	*CpTI*	Insect tolerance to *Pieris rapae* L.	N/A	Hongjun et al. (1997)
Hercules, Brunswick, Cape spitz, Copenhagen	*A. tumefaciens* (LBA4404)	*nptII*	Resistance to kanamycin	Cotyledons	Pius and Achar (2000)
Scorpio, Testie	*A. tumefaciens* (EHA105)	*cry1Ia3*	Resistance to diamond back moth (*P. xylostella*)	Hypocotyls, cotyledons, petioles	Jin et al. (2000)
Uji	*A. tumefaciens* (LBA4404)	*GO*	Enhanced tolerance to black rot disease by *Xanthomonas campestris* pv. *campestris*	Hypocotyls	Lee et al. (2002)
*B. oleracea var. capitata* cv. N/A	*A. tumefaciens* (LBA4404)	*BcA9*	Induction of male sterile cabbage	Hypocotyls	Lee et al. (2003)
*Golden Acre*	*A. tumefaciens* (GV2260)	*bet*A	Enhanced salt tolerance	Hypocotyls	Bhattacharya et al. (2004)
DTC 507	*A. tumefaciens* (N/A)	*cry1 b*	Resistance to diamond back moth (*P. xylostella*)	Hypocotyls, cotyledonary nodes	Paul et al. (2005)
Xiaguang	*A. tumefaciens* (LBA4404)	*vhb*	Increases submergence tolerance	Hypocotyls, cotyledons, petioles	Li et al. (2005)
N/A	*A. tumefaciens* (LBA4404)	*OC*-*I*	Insect resistance	Hypocotyls, cotyledons	Lei et al. (2006)
P34I5, P22I5	*A. rhizogenes* (A4M70GUS)	*gus*	N/A	Hypocotyls, cotyledons	Stretnović-Rajičić et al. (2006)
Summer Summit, KY cross	Biolistic method	*gus*	N/A	Chloroplasts	Liu et al. (2007)
Summer Summit, KY cross	Biolistic method	*cry1Ab*	Resistance to diamond back moth (*P. xylostella*)	Chloroplasts	Liu et al. (2008)
KY Cross	*A. tumefaciens* (GV2260)	*AtHSP101*	Increase the high temperature tolerance	Hypocotyls, shoot tip segments	Rafat et al. (2010)
CA21-3	*A. tumefaciens* (EHA105)	*cry1Ba3*	Resistance to diamond back moth (*P. xylostella*)	Hypocotyls, cotyledons	Deng-xia et al. (2011)
A21-3’	*A. tumefaciens* (LBA4404)	*cry1Ia8, cry1Ba3*	Resistance to diamond back moth (*P. xylostella*)	Hypocotyls	Yi et al. (2013)
N/A	Biolistic method		N/A	Chloroplasts	Tseng et al. (2014)
AD BENTAM	*A. tumefaciens* (LBA4404)	*PMI*, *JMT*	PMI/mannose selection system; resistance to stress	Hypocotyls, cotyledons	Hur and Min (2015)
Pride of India	*A. tumefaciens* (N/A)	*cry IAa*	Study of the effect of antibiotic sensitivity on cabbage tissue	Hypocotyls, cotyledons	Gambhir et al. (2017b)

*N/A* not available

### Crucial factors for successful *Agrobacterium*-mediated cabbage transformation

Successful genetic transformation via *A. tumefaciens* depends on susceptibility of cabbage infections and GOI incorporation to the genome as well as ability to regenerate plants from transformed cells in in vitro culture. The results from the last three decades allowed to identify factors which played a pivotal role in the successful transformation of cabbage.

### Explant type and explant age

Different cabbage explants were used for transformation; however, hypocotyl and shoot tip derived ones offered the best transformation efficiency (Rafat et al. 2010). Young explants, i.e., 4- to 9-day-old seedlings were mainly used for transformation (Li et al. 2005; Rafat et al. 2010; Yi et al. 2013). As mentioned previously, such explants were characterized with better morphogenic response and had more flexible cell wall which made them more susceptible to PGRs and *Agrobacterium* impact. Consequently, it is recommended to place explants on a pre-culture medium with PGR as an osmotic practice (Yi et al. 2013; Hur and Min 2015). Interestingly, not every type of cabbage explant requires such treatment. The pre-culture treatment is indispensable in the case of hypocotyl or cotyledon explants hypersensitive to *Agrobacterium,* while it is not necessary for shoot tip explants as the studies of Rafat et al. (2010) and Hur and Min (2015) revealed.

### Agrobacterium strain, concentration, inoculation and co-cultivation period

Summarizing the examples of successful cabbage transformation (Table 2), it was noticed that *Agrobacterium* LBA 4404 octopine strain was commonly used. It is known that virulence of a strain is important for successful transformation and the aforementioned strain is of moderate virulence. *Agrobacterium* concentration (OD_600_) was one of the most crucial factors for plant infection. According to the available data, *Agrobacterium* at the concentrations 0.4–0.5 or 1.6–1.8 was used for *B. oleracea* var. *capitata* transformation (Metz et al. 1995; Li et al. 2005; Rafat et al. 2010; Yi et al. 2013). Based on the results it can be stated that, to avoid explants necrosis or even death, *Agrobacterium* OD at ƛ = 600 nm should be adjusted to 0.05–0.1, the inoculation time should not exceed 10 min and co-cultivation time—3 days (Yi et al. 2013). Furthermore, supplementation of the bacterial growth medium or the co-cultivation medium with phenolic compounds was reported to significantly improve transformation efficiency regardless of the *Agrobacterium* strain used (Bhattacharya et al. 2002; Rafat et al. 2010).

### Antibiotics

One of the indispensable factors during the genetic transformation of plants is the use of specific antibiotics (e.g., selective or bactericidal ones). Their concentration should be optimized before transformation to determine an effective dose leading to plants regeneration as well as a lethal dose for *Agrobacterium*. This step is extremely important due to the fact that the prolonged presence of *Agrobacterium* in plant tissues affects the growth and development of explants and can even cause necrosis and consequently their death (Stanišić et al. 2018). Furthermore, presence of *Agrobacterium* in tissue of putative transformants could result in false positive results in the molecular analyses. In addition, elimination of *Agrobacterium* from transformed plants prevents the possibility of accidental release of transgenes to the environment when transformants are transferred to the soil (Tambarussi et al. 2015). Beta-lactam antibiotics (e.g., carbenicillin, cefotaxime and timentin) are most commonly used for combating *Agrobacteria* after genetic transformation (Stanišić et al. 2018). Due to the difficulty in the transformation of cabbage, only few papers related to this subject were published. However, in the articles concerning *Agrobacterium*-mediated transformation of cabbage, mainly cefotaxime or carbenicillin (as a bacterial eliminating factor) at 500 mg/L concentration was used along with kanamycin (25 mg/L) (a selection factor) (Paul et al. 2005; Rafat et al. 2010; Yi et al. 2011).

According to the author’s knowledge, only few publications exist on the optimization of antibiotic concentrations after the genetic transformation of cabbage. In the last decade, only two publications strictly related to this topic appeared. Rafat et al. (2010) in his research tested different variants of hygromycin as a selection factor; however, he did not provide results from this part of the experiment. The only conclusion about hygromycin was that it definitely had a stressful effect on transformed plants. Whereas Gambhir et al. (2017b) focused on the effect of cefotaxime and kanamycin in a wide range of concentrations on transformed cabbage tissues. Their results revealed a negative correlation between the concentration of kanamycin (0–60 mg/L) and fresh weight of the explants (leaf and petiole), while cefotaxime at different concentrations (0–500 mg/L) did not have much effect on cabbage regeneration potential.

## Particle bombardment of cabbage

Since the first stable chloroplast genetic transformation in higher plants was proved, it has been successfully exploited to transform a wide range of different species of plants (Hnatuszko-Konka et al. 2014; Bock 2015; Gerszberg and Hnatuszko-Konka 2017). Generally, it is known that there is a high level of foreign gene expression in plastids, thus the protein level of these transgenes is high. Therefore, this transformation method can be another way to obtain transgenic cabbage with quite new agronomic or horticultural traits. The first protocol for stable chloroplast transformation in *B. oleracea* var. *capitata* by particle bombardment was established by Liu et al. (2007). Based on this procedure, a year later Liu et al. (2008) obtained transgenic cabbage with expression of *cry1Ab* gene in chloroplasts. However, the transformation frequency for the same cultivars (K–Y and Summer Summit) was lower than in the previous study (Liu et al. 2007). It might have resulted from the fact that fully expanded leaves were used as explants. Although older leaves have mature chloroplasts, it is more difficult to obtain transgenic plants (transplastomic lines) from them. Liu et al. (2008) pointed out that successful foreign gene integration with chloroplast genome of cabbage required flanking sequence homology in the applied vector to the chloroplast genome. Extremely low homology (less than 100%) is the cause of low transformation frequency (Tseng et al. 2014).

## Conclusions

*B. oleracea* var. *capitata* as a crop plant is of great economic importance all over the world. Unfortunately, its yields are depleted by various environmental stresses (abiotic and biotic). However, great advance in the field of plant tissue culture, molecular biology as well as genetic engineering has offered new solutions for genetic improvement of significant vegetable crops including cabbage. This review provides an insight into different regeneration and transformation studies in cabbage. Based on the literature data, it can be concluded that *B. oleracea* var. *capitata* appears recalcitrant to genetic engineering. Moreover, its successful regeneration and transformation procedure strongly depends on the genotype. A lot of factors were proved essential for increasing shoot regeneration and for enhancing transformation frequency. To obtain the best effects, it is necessary to establish optimized procedures mentioned above, even for particular important varieties. If this condition is met, it will be possible to commercialize cabbage plants with new desirable traits. Therefore, according to the author’s conviction, the proposed review of the available data could be a useful starting point for the development of efficient transformation/regeneration protocols for specific cultivars.

### *Author contribution statement*

AG conceived the idea of the review and prepared the initial outline, tables, figures as well as wrote the manuscript.
